# Association of Estrogen Receptor Genes Polymorphisms With Polycystic Ovary Syndrome: A Systematic Review and Meta-Analysis Based on Observational Studies

**DOI:** 10.3389/fendo.2021.726184

**Published:** 2021-10-04

**Authors:** Siyu Zhou, Shu Wen, Yongcheng Sheng, Meina Yang, Xiaoyang Shen, Yan Chen, Deying Kang, Liangzhi Xu

**Affiliations:** ^1^ Department of Obstetrics and Gynaecology, West China Second University Hospital, Sichuan University, Chengdu, China; ^2^ Key Laboratory of Birth Defects and Related Diseases of Women and Children, Sichuan University, Ministry of Education, Chengdu, China; ^3^ Reproductive Endocrinology and Regulation Laboratory, West China Second University Hospital, Sichuan University, Chengdu, China; ^4^ Department of Evidence-based Medicine and Clinical Epidemiology, West China Hospital, Sichuan University, Chengdu, China; ^5^ Center of Biostatistics, Design, Measurement and Evaluation (CBDME), West China Hospital, Sichuan University, Chengdu, China

**Keywords:** polycystic ovary syndrome, polymorphisms, estrogen receptor, meta-analysis, gene variants

## Abstract

**Purpose:**

Controversial results existed in amounts of studies investigating the authentic association of estrogen receptor genes (ESR1 and ESR2) polymorphisms with the occurrence and progression of polycystic ovary syndrome (PCOS). The inconsistency might result from different loci, sample sizes, and ethnicities. To find the potential correlations between ESR1/ESR2 polymorphisms and PCOS risk, we conducted the first systematic review and meta-analysis to comprehensively summarize current studies in a large combined population.

**Methods:**

Eligible studies were retrieved from PubMed, MEDLINE, EMBASE, Cochrane Library, CBM, CNKI, WANFANG, and VIP up to February 28, 2021. The quality of studies was assessed using the Newcastle–Ottawa Scale (NOS) scoring system. Odds ratios (ORs) and 95% confidence intervals (95%CIs) were calculated to synthesize data in five genetic models. Subgroup analyses were conducted by ethnicity. Heterogeneity and publication bias were also assessed. The protocol was registered in PROSPERO under the number CRD42021239200.

**Results:**

A total of 8 studies involving 1,522 PCOS patients and 4,198 controls were included. No evidence demonstrated the association of ESR1 rs2234693 (OR=1.07 95%CI 0.98–1.18), ESR1 rs9340799 (OR=0.99 95%CI 0.69–1.43), or ESR2 rs4986938 (OR=1.06 95%CI 0.81–1.38) polymorphisms and PCOS risk in five genetic models. According to stratified subgroup analyses, ethnicity was considered the major source of heterogeneity. No publication bias was found in eligible studies.

**Conclusion:**

The present meta-analysis found no significant associations between the variants of ESR1 rs2234693, ESR1 rs9340799, ESR2 rs4936938, and individual PCOS susceptibility, even if ethnicity was taken into account.

**Systematic Review Registration:**

The protocol was registered in PROSPERO (available from https://www.crd.york.ac.uk/PROSPERO) with the ID number CRD42021239200.

## Introduction

Although the pathogenesis and etiology of polycystic ovary syndrome (PCOS) remain hitherto unelucidated, it is widely considered multifactorial, that is, both environmental exposure and genetic susceptibility interact with each other and jointly influence PCOS incidence (1). To date, numerous studies suggested that genetic polymorphisms contribute substantially to the development of PCOS; over 100 genes are candidates, which functioned mainly in steroid biosynthesis, steroid action, glucose metabolism, and lipid metabolism (2–7). Rising interests were drawn in abnormal function of female sex hormone and hormonal receptors of PCOS women, and the impact of the mutation of some critical genes in the hormone metabolism pathways such as androgen receptor (AR) (8), luteinizing hormone receptor (LHR) (9), and follicle-stimulating hormone receptor (FSHR) (10) has been investigated in some meta-analyses. It was found that they were associated with some characteristics of PCOS such as clinical and/or biochemical hyperandrogenism, ovulatory dysfunction, and polycystic ovaries. However, the studies on estrogen receptor genes (ESR) were still controversial.

Estrogen mediates ovarian folliculogenesis and ovulation by binding to estrogen receptor α (ERα) and estrogen receptor β (ERβ), class 1 members of the superfamily of nuclear hormone receptor protein (11), which plays a prominent role in regulating cellular proliferation, differentiation, and apoptosis (12). Furthermore, two subtypes of ESR are nowadays identified, designated as ESR1 and ESR2, encoding ERα and ERβ separately. The ESR1 gene is located on chromosome 6q25.1, encompassing 140 kDa of DNA composed of 8 exons, and its introns are highly conserved (13). The ESR2 gene is located on chromosome 14q23.1, which contains 9 exons. Previous genetically engineered researches of knockout mice have revealed an interesting phenomenon that the absence of ESR1 can be associated with PCOS symptoms of irregular estrous, infertile, higher androgen level, and the formation of hemorrhagic follicles (14). Besides, the absence of ESR2 can lead to more early atretic follicles and fewer corpora lutea, suggesting partially follicular developmental and mature obstruction (15). Therefore, polymorphisms in the steroid action genes ESR1 and ESR2 may contribute to individual susceptibility to PCOS.

In recent years, an increasing number of investigations have focused on the potential association of ESR1 and ESR2 polymorphisms with PCOS susceptibility. Nonetheless, some studies held the view that these gene polymorphisms of ESR1 and ESR2 were positively related to PCOS (16, 17), whereas other studies reported no relation (18–22) or negative relation (23). The results remain inconsistent presumably due to loci and insufficient sample sizes in single study (16–23). Besides, different allelic frequencies between ethnic groups were the most crucial reason leading to long-existed controversy; thus, scientific and rigorous meta-analysis was required to summarize current studies. In this systematic review and meta-analysis, we aim to better clarify the relationship between single nucleotide polymorphisms (SNPs) in ESR1/ESR2 and the risk of PCOS in a large combined population by comprehensively collecting the existing data.

## Materials and Methods

### Identification of Eligible Studies

A search of literature was conducted in the PubMed, MEDLINE, EMBASE, Cochrane Library, CBM, CNKI, WANFANG, and VIP. The search period covered the start of the databases to February 28, 2021. The search strategy used to identify eligible studies included (polycystic ovary syndrome OR polycystic ovary OR PCOS OR PCO) and (Single Nucleotide Polymorphism OR SNP OR gene variant OR mutation OR genotype) and (estrogen receptor OR ESR1 OR ERα OR ESR2 OR ERβ), without language restriction. All references cited by these studies were manually screened in order to identify relevant studies.

### Inclusion and Exclusion Criteria

Studies were included if they met all of the following criteria: (1) case-control studies, cross-sectional studies, case series, and cohort studies that were published in peer-reviewed journals; (2) PCOS patients diagnosed with one of the following criteria: the National Institutes of Health (NIH) (24), Rotterdam criteria (25), or the Androgen Excess and PCOS Society (AE-PCOS Society) criteria (26); and (3) the sample size and genotype frequency of case and control groups were directly provided or original data were sufficient to calculate the odds ratios (ORs) and 95% confidence intervals (95% CIs). Studies were excluded if they (1) were conference abstracts or reviews with non-extractable data; (2) deviated from Hardy–Weinberg equilibrium (HWE); or (3) recruited participants from one family. If data based on the same population were reported by different authors or research center, only the most comprehensive publication was included for meta-analysis.

### Data Extraction

The titles and abstracts were scrutinized, and the data of included studies were extracted from fully retrieved texts by two investigators independently (SZ and SW). The characteristics of eligible studies were extracted according to Cochrane guidelines. The following contents were extracted: first author, year of publication, country/ethnicity, genotyping method, PCOS diagnostic criteria, the characteristics of cases and controls, HWE, and genotype frequency in cases and controls for ESR1 and ESR2 polymorphisms, respectively. Dissents were noted and resolved by discussing with the third investigator (LX).

### Methodological Quality Assessment

The Newcastle–Ottawa scoring system (NOS), recommended by the Cochrane Working Group, was conducted to assess the quality of these nonrandomized studies (27). Evaluating concerns of NOS consists of the following three aspects: selection of subjects, comparability of enrolments, and outcome of interest ascertainment. The total score ranged from 0 to 9. Studies with a score from 0 to 3, 4 to 6, and 7 to 9 were considered to be of low, moderate, and high quality, respectively. Evaluation of evidence quality was determined by two reviewers (SZ and SW) independently, with any disagreement resolved by the discussion (SZ, SW, and LX).

### Statistics Analysis

The HWE was evaluated for each study with a chi-square (χ²) test in the control group by the IBM SPSS statistics 25 software. P> 0.05 indicated that the study population was in HWE. The 95%CI and OR were utilized to evaluate the association strengths of the SNPs of ESR1/ESR2 with susceptibility to PCOS. And five genetic models (allele model, dominant model, recessive model, heterozygote model, and homozygote model) were conducted to evaluate the influence of the genotype. The Q-test was used for heterogeneity testing, and the inconsistency index (I²), a test statistic, was used for quantification. In the Q test, when I² ≤ 50%, the fixed-effects model was established to analyze. Otherwise, the random-effects model was performed. Next, a Z-test was performed to evaluate the statistical significance of combined ORs in the quantitative synthesis. When P value < 0.05, it was considered a statistically significant association between SNP mutation and PCOS. Subgroup analyses were stratified by ethnicity, which might be the potential source of inconsistencies. Funnel plots of the outcomes were used to detect publication bias; an asymmetric graph suggested possible publication bias. Egger’s regression test was also applied to assess the publication bias of selected studies, and as proposed by Egger, P value < 0.10 indicated significant publication bias. Sensitivity analysis was performed where appropriate to determine the robustness of the results. All analyses were carried out in the Review Manager software (Version 5.3; Copenhagen: The Nordic Cochrane Centre, The Cochrane Collaboration, 2014) and the STATA software (Version 13.0; Stata Corporation, College Station, TX).

## Results

### Study Selection

**Figure 1:**
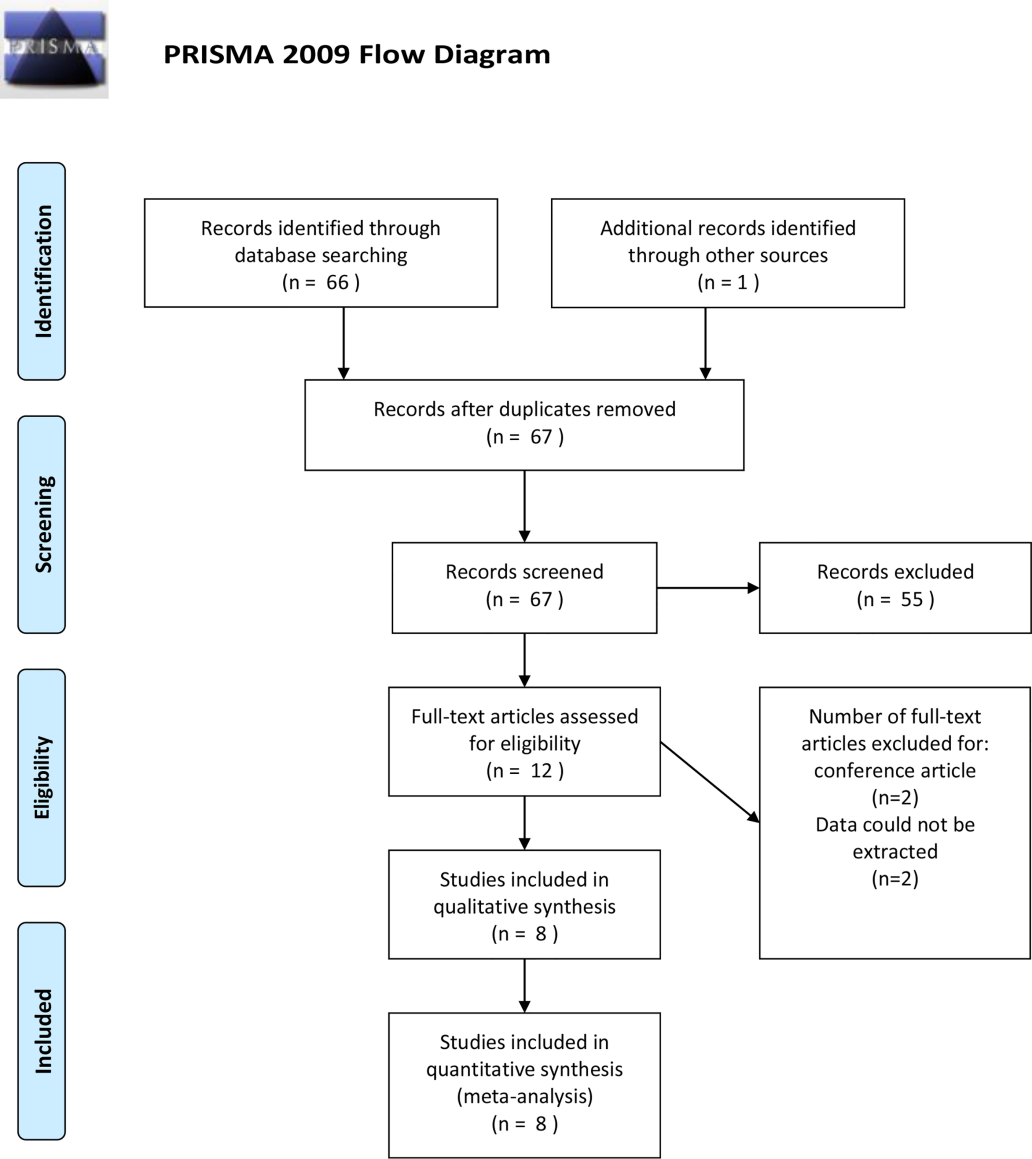
PRISMA flow diagram of study selection for the systematic review and meta-analysis.

A total of 67 potential citations were acquired from electronic databases after exclusion of duplicate articles. Of these, by scanning titles and abstracts carefully, 55 records were removed for irrelevant themes. The remained 12 articles were full-text screened, and another 4 records were excluded subsequently, among which 2 were conference abstracts from the same population, 1 was retracted, and 1 reported non-extractable data. We contacted the corresponding authors for further data; nonetheless, no reply was received. Finally, eight eligible case-control studies that met our criteria were included in the qualitative and quantitative synthesis (16–23). The selection process was documented with a flowchart of Preferred Reporting Items for Systematic Reviews and Meta-analyses (PRISMA) (**Figure 1**).

### Characteristics and Methodological Quality of Included Studies

The characteristics of each eligible study are listed in **Table 1**. A total of 5,720 subjects were reported in 8 studies, including 1,522 PCOS patients and 4,198 controls. These studies, which ranged from 2001 to 2018, spanned seven countries and involved two ethnicities: Asian and Caucasian. Despite the fact that these studies explored the association between multiple SNPs in ESR1/ESR2 and PCOS, most SNPs were reported only once. Therefore, the SNPs that were examined in more than three populations were selected for ultimate pooled analysis. The PCOS patients and control people were genotyped for ESR1 rs2234693 in six studies (16, 17, 19, 20, 22, 23), for ESR1 rs9340799 in five studies (16, 19, 20, 22, 23), and for ESR2 rs4986938 in six studies (16–19, 21, 22). The genotype distributions were in agreement with HWE; the age and body mass index (BMI) were comparable between patients and controls in most studies as reported except that two studies (21, 22) did not record the BMI of the participants. The scores of included studies assessed by NOS were ranged from 5 to 8 (**Table 2**), which means all included articles were regarded as moderate or high quality.

**Table 1:** Characteristics of included studies.

First author	Year	Country/Ethnicity	Genotyping method	PCOS diagnostic criterion	Number of participants(case/control)	Age, years(case/control,mean ± SD)	BMI, kg/m^2^(case/control,mean ± SD)	P for HWE	Source of control	Gene polymorphism	Biomedical and clinical data
Jiao (23)	2018	China/Asian	PCR	NIH	361/331	28.1 ± 3.7/28.4 ± 4.2	24.9 ± 4.3/21.5 ± 3.4	0.69	Hospital	ESR1:rs1709183,rs2228480,rs2234693,rs3020314,rs3778082,rs3778099,rs3798573,rs3798577,rs851982,rs9322331,rs9340799,rs1999805 ESR2:rs1255998,rs928554,rs960070,rs1152579,rs944459	Age, BMI, LH, FSH, LH/FSH, T, E2
Khafagi (17)	2014	Iran/Caucasian	PCR	Rotterdam	100/100	29.4 ± 4.9/30.8 ± 3.9	26.9 ± 5.04/24.3 ± 4.6	0.73	Hospital	ESR1: rs2234693 ESR2:rs4986938	Age, BMI, FSH, LH, E2, Cycle length
Kim (18)	2010	Korea/Asian	PCR	Rotterdam	138/290	26.1 ± 5.5/33.8 ± 4.5	23.0 ± 4.5/21.5 ± 3.3	reported >0.05	Hospital and community	ESR2:rs4986938	WHR, Hirsutism score, TT,FT, 17-OHP, SHBG, DHEAS, LH, FSH, LH/FSH, E2, Fasting plasma glucose, Fasting insulin, HOMA-IR, Postprandial 2-hour glucose, Postprandial 2-hour insulin
Liaqat (16)	2015	Pakistan/Asian	PCR	Rotterdam	96/96	26.9 ± 4.4/26.0 ± 3.5	31.1 ± 1.47/30.5 ± 1.66	0.85	Community	ESR1:rs2234693,rs9340799,rs8179176 ESR2:rs4986938	WHR, Menarche, Gynecological history, Patient symptoms
Nectaria (19)	2012	Greece/Caucasian	PCR	–	180/140	23.7 ± 6.4/24.8 ± 6.9	26.6 ± 6.9/20.9 ± 1.8	0.70	–	ESR1:rs2234693,rs9340799 ESR2:rs1256049,rs4986938	LH/FSH, SHBG, FAI,TT, DHEAS, Fasting glucose/insulin ratio
Silva (20)	2015	Brazil/Caucasian	PCR	Rotterdam	99/104	30.7 ± 5.2/29.1 ± 7.9	29.5 (8.0)/22.9 (5.1)	0.57	Community	ESR1:rs2234693,rs9340799	WC, LAP, fasting glucose, T, CRP, FSH, postload glucose
Sundarrajan (21)	2001	China/Asian	PCR	–	30/150	25.6 ± 6.7/32.7 ± 4.6	-/-	reported >0.05	Community	ESR2:rs1256049,rs4986938	
Valkenburg (22)	2011	Netherland/Caucasian	Taqman	Rotterdam	518/2996	28.7 ± 4.96/-	26.2 ± 4.96/-	0.38	Community	ESR1:rs2234693,rs9340799 ESR2: rs4986938	WC, LH, FSH, E2, P, 17-OHP, T, SHBG, FAI, Androstenedione, DHEA, DHEAS, Cortisol, Fasting glucose, Fasting insulin, HOMA-IR

PCOS, polycystic ovarian syndrome; SD, standard deviation; BMI, body mass index; HWE, Hardy–Weinberg equilibrium; PCR, polymerase chain reaction; NIH, National Institutes of Health; ESR, estrogen receptor; LH, luteinizing hormone; FSH, follicle-stimulating hormone; T, testosterone; E2, estradiol; WHR, waist/hip ratio; TT, total testosterone; FT, free testosterone; 17-OHP, 17α-hydrooxyprogesterone; SHBG, sex hormone binding globulin; DHEAS, dehydroepiandrosterone sulfate; HOMA-IR, homeostatic model assessment for insulin resistance; FAI, free androgen index; WC, waist circumference; LAP, lipid accumulation product; CRP, C-reactive protein; P, progesterone; DHEA, dehydroepiandrosterone.

**Table 2:** Methodological quality assessment of included studies.

First author	Year	selection				Comparability of cases and controls on the basis of the design or analysis^a^	Exposure			Total score
		Adequate definition of the cases	Representativeness of the cases	Selection of Controls	Definition of Controls		Ascertainment of exposure	Same method of ascertainment for cases and controls	Non-response rate	
Jiao (23)	2018	☆	☆		☆	☆☆	☆	☆	☆	8
Khafagi (17)	2014	☆			☆	☆☆		☆		5
Kim (18)	2010	☆	☆		☆	☆☆	☆	☆	☆	8
Liaqat (16)	2015			☆	☆	☆☆	☆	☆	☆	7
Nectaria (19)	2012				☆	☆☆		☆	☆	5
Silva (20)	2015	☆	☆	☆	☆	☆☆		☆	☆	8
Sundarrajan (21)	2001			☆	☆	☆	☆	☆		5
Valkenburg (22)	2011		☆	☆	☆	☆		☆	☆	6

a A maximum of two stars can be allotted in this category: one for ethnicity, the other for other controlled factors. One star represents one point for this item.

### Associations Between ESR1 rs2234693 Polymorphism and PCOS Susceptibility

**Figure 2:**
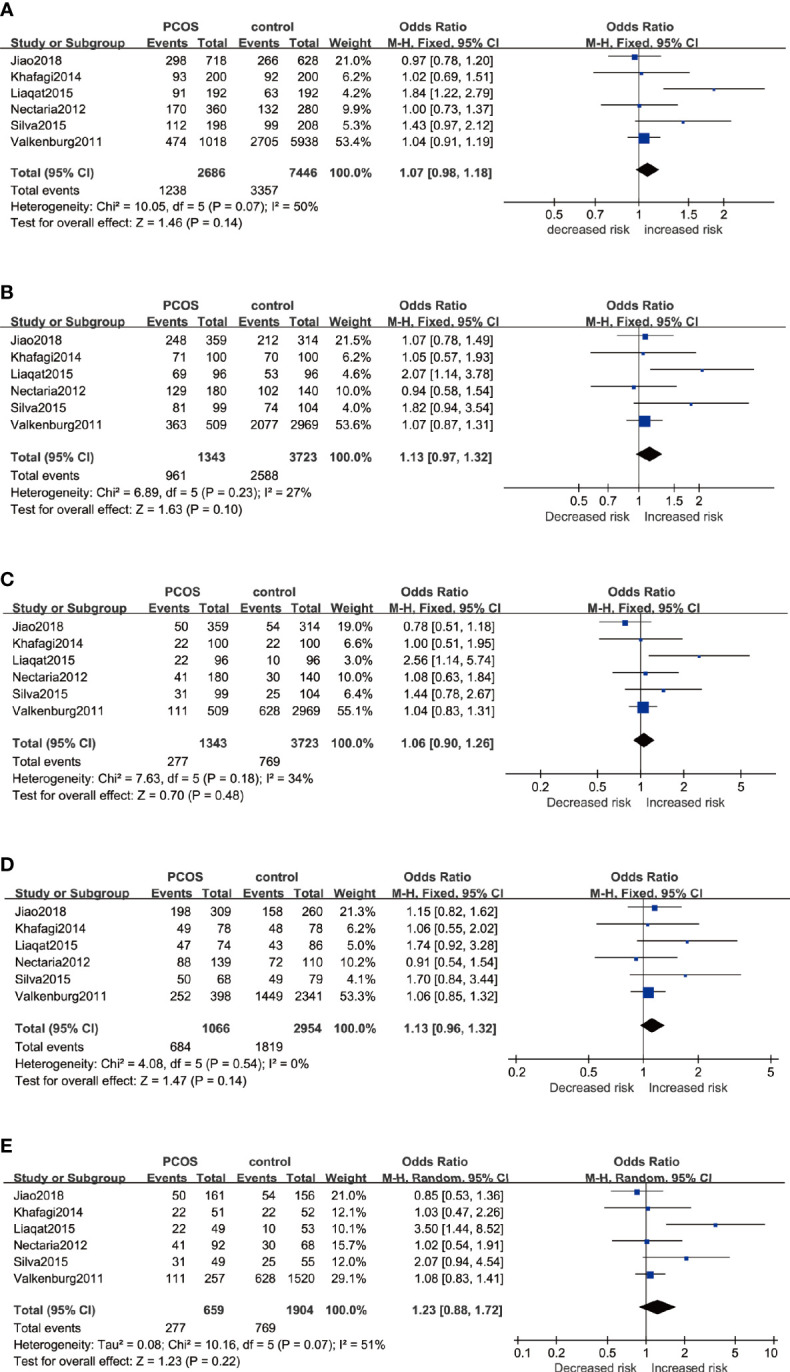
Forest plots of the association between the ESR1 rs2234693 gene and risk of PCOS using different genetic models in overall analysis: **(A)** Allele model (C *versus* T), **(B)** Dominant model (CC+TC *versus* TT), **(C)** Recessive model (CC *versus* TT+TC), **(D)** Heterozygote model (TC *versus* TT), and **(E)** Homozygote model (CC *versus* TT). In each model, solid squares represent the OR, horizontal lines represent 95%CI, and diamond represents the pooled OR and 95%CI. OR, odds ratios; 95% CI, 95% confidence intervals; I^2^, inconsistency index.

Six studies that recruited a total of 1,354 PCOS patients and 3,767 controls were measured for rs2234693. The analyses were conducted in five genetic models, and the results are presented in **Figure 2**. The Q-test and I^2^ statistic indicated that mild heterogeneity was revealed across these studies. Ultimately, no significant association was identified in five genetic models: Allele model (C *versus* T): OR=1.07 95%CI 0.98–1.18, I^2^ = 50%; Dominant model (CC+TC *versus* TT): OR=1.17 95%CI 0.96–1.42, I^2^ = 27%; Recessive model (CC *versus* TT+TC): OR=1.06 95%CI 0.90–1.26, I^2^ = 34%; Heterozygote model (TC *versus* TT): OR=1.13 95%CI 0.96–1.32, I^2^ = 0%; Homozygote model (CC *versus* TT): OR=1.23 95%CI 0.88–1.72, I^2^ = 51%. Besides, subgroup analyses were performed based on a predefined factor: ethnicity. In Asians, to reduce the influence of severe heterogeneity, the random-effects model was conducted in most genetic models except for the heterozygote model, which showed no significant statistical heterogeneity. In Caucasians, there was no between-study heterogeneity, due to which the fixed-effects model was applied. Likewise, there was no significant association between rs2234693 and PCOS susceptibility in Asians or Caucasians (**Table 3**).

**Table 3:** Summary ORs in the meta-analysis.

	No. of studies	OR (95%CI)	I^2^ (%)	OR (95%CI)	I^2^ (%)	OR (95%CI)	I^2^ (%)	OR (95%CI)	I^2^ (%)	OR (95%CI)	I^2^ (%)
**rs2234693**		C/T		CC+TC/TT		CC/TT+TC		TC/TT		CC/TT	
Overall	6	1.07 (0.98-1.18)	50	1.17 (0.96-1.42)	27	1.06 (0.90-1.26)	34	1.13 (0.96-1.32)	0	1.23 (0.88-1.72)	51
Ethnicity											
Asian	2	1.30 (0.69-2.45)	86	1.42 (0.75-2.68)	72	1.02 (0.71-1.47)	85	1.26 (0.94-1.70)	21	1.64 (0.41-6.54)	87
Caucasian	4	1.06 (0.95-1.19)	0	1.09 (0.91-1.30)	0	1.07 (0.89-1.30)	0	1.08 (0.89-1.30)	0	1.13 (0.90-1.41)	0
**rs9340799**		G/A		GG+AG/AA		GG/AA+AG		AG/AA		GG/AA	
Overall	5	0.99 (0.69-1.43)	89	0.99 (0.60-1.62)	88	1.05 (0.71-1.56)	52	0.96 (0.60-1.53)	86	1.05 (0.60-1.84)	72
Ethnicity											
Asian	2	0.91 (0.28-2.99)	96	0.87 (0.20-3.82)	95	1.04 (0.25-4.27)	86	0.81 (0.21-3.05)	93	1.01 (0.14-7.52)	92
Caucasian	3	1.08 (0.96-1.22)	0	1.09 (0.92-1.29)	0	1.14 (0.89-1.45)	0	1.07 (0.89-1.28)	0	1.18 (0.91-1.53)	0
**rs4986938**		A/G		AA+GA/GG		AA/GG+GA		GA/GG		AA/GG	
Overall	6	1.06 (0.81-1.38)	70	1.10 (0.77-1.57)	72	1.08 (0.87-1.35)	0	1.07 (0.75-1.53)	68	1.10 (0.87-1.40)	28
Ethnicity											
Asian	3	1.11 (0.48-2.57)	87	1.15 (0.43-3.07)	87	1.84 (0.90-3.76)	5	1.10 (0.42-2.87)	84	2.06 (1.00-4.26)	44
Caucasian	3	1.01 (0.89-1.14)	0	1.00 (0.85-1.19)	20	1.02 (0.81-1.30)	0	1.00 (0.83-1.19)	30	1.02 (0.79-1.32)	0

OR, odds ratios; 95% CI, 95% confidence intervals; I^2^, inconsistency index.

### Associations Between ESR1 rs9340799 Polymorphism and PCOS Susceptibility

**Figure 3:**
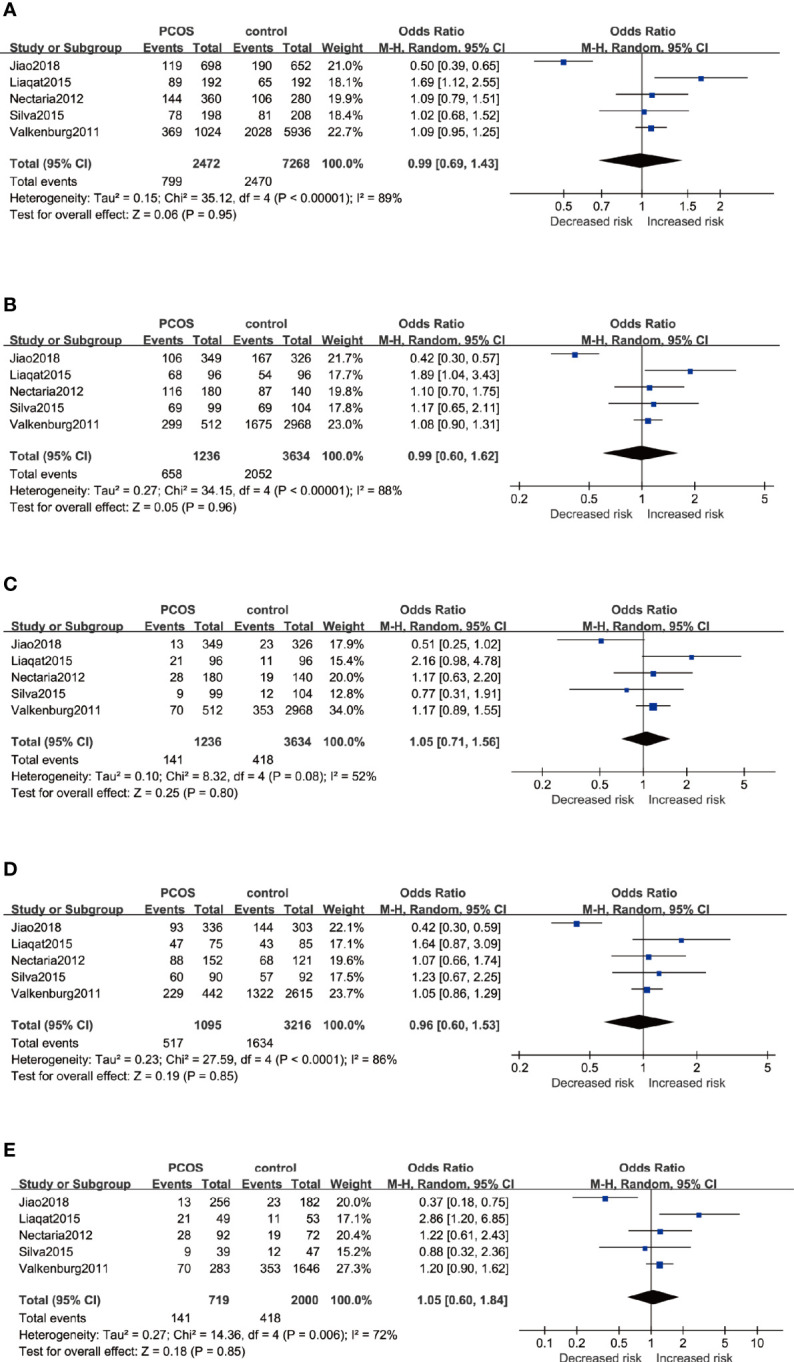
Forest plots of the association between the ESR1 rs9340799 gene and risk of PCOS using different genetic models in overall analysis: **(A)** Allele model (G *versus* A), **(B)** Dominant model (GG+AG *versus* AA), **(C)** Recessive model (GG *versus* AA+AG), **(D)** Heterozygote model (AG *versus* AA), and **(E)** Homozygote model (GG *versus* AA). In each model, solid squares represent the OR, horizontal lines represent 95%CI, and diamond represents the pooled OR and 95%CI. OR, odds ratios; 95% CI, 95% confidence intervals; I^2^, inconsistency index.

By pooling five eligible studies that consisted of 1,254 cases and 3,667 controls, no significant association was observed in five genetic models, respectively as depicted in **Figure 3**: Allele model (G *versus* A): OR=0.99 95%CI 0.69–1.43, I^2^ = 89%; Dominant model (GG+AG *versus* AA): OR=0.99 95%CI 0.60–1.62, I^2^ = 88%; Recessive model (GG *versus* AA+AG): OR=1.05 95%CI 0.71–1.56, I^2^ = 52%; Heterozygote model (AG *versus* AA): OR=0.96 95%CI 0.60–1.53, I^2^ = 86%; Homozygote model (GG *versus* AA): OR=1.05 95%CI 0.60–1.84, I^2^ = 72%. With severe heterogeneity presented, the random-effects model was applied in every genetic model to synthesize the OR and 95% CI. In subgroup analyses, noticeable heterogeneity was revealed in the Asian population; thus, the random-effects model was chosen for two studies based on Asians. No heterogeneity was found in studies based on Caucasians in which the fixed-effects model was selected. Nonetheless, no statistical association was found in the subgroups of Asians or Caucasians irrespective of whether allele, dominant, recessive, heterozygote, or homozygote comparison of inheritance was used (**Table 3**).

### Associations Between ESR2 rs4986938 Polymorphism and PCOS Susceptibility

**Figure 4:**
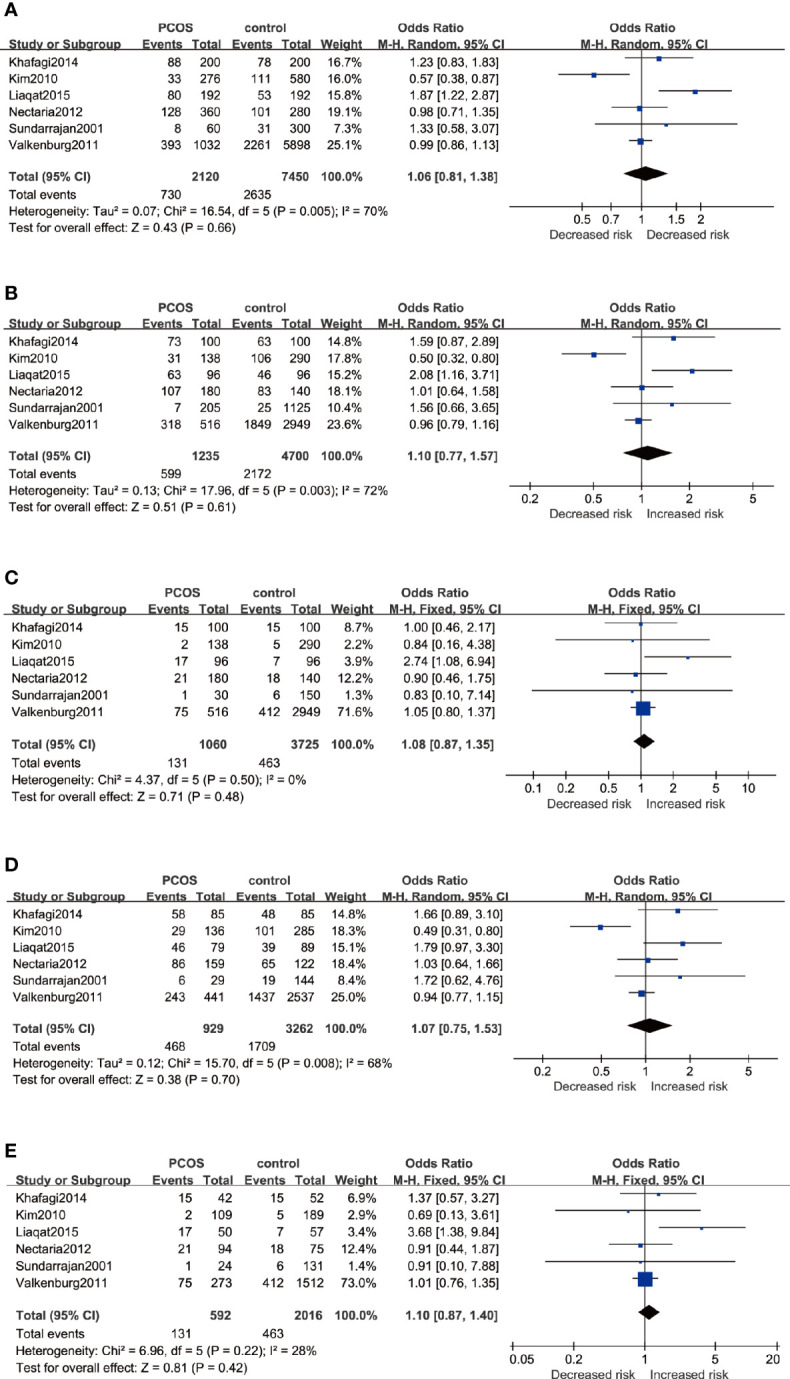
Forest plots of the association between the ESR2 rs4986938 gene and risk of PCOS using different genetic models in overall analysis: **(A)** Allele model (A *versus* G), **(B)** Dominant model (AA+GA *versus* GG), **(C)** Recessive model (AA *versus* GG+GA), **(D)** Heterozygote model (GA *versus* GG), and **(E)** Homozygote model (AA *versus* GG). In each model, solid squares represent the OR, horizontal lines represent 95%CI, and diamond represents the pooled OR and 95%CI. OR, odds ratios; 95% CI, 95% confidence intervals; I^2^, inconsistency index.

The association between ESR2 rs4986938 and PCOS risk was assessed in six pieces of research performed in different countries, involving 1,062 PCOS women and 3,772 healthy women. Prominent heterogeneity was revealed in the allele model, the dominant model, and the heterozygote model in which meta-analysis was performed with a random-effects model, while the fixed-effects model was adopted in the other two genetic models. The results of meta-analysis suggested that ESR2 rs4986938 was not associated with increased or decreased risk of PCOS (**Figure 4**): Allele model (A *versus* G): OR=1.06 95%CI 0.81–1.38, I^2^ = 70%; Dominant model (AA+GA *versus* GG): OR=1.10 95%CI 0.77–1.57, I^2^ = 72%; Recessive model (AA *versus* GG+GA): OR=1.08 95%CI 0.87–1.35, I^2^ = 0%; Heterozygote model (GA *versus* GG): OR=1.07 95%CI 0.75–1.53, I^2 =^ 68%; Homozygote model (AA *versus* GG): OR=1.10 95%CI 0.87–1.40, I^2^ = 28%. Subsequently, subgroup analyses were conducted in Asians with high heterogeneity (random-effects model) and Caucasians with low heterogeneity (fixed-effects model). No association was found to be significant in different ethnicities (**Table 3**).

### Sensitivity Analysis

**Figure 5:**
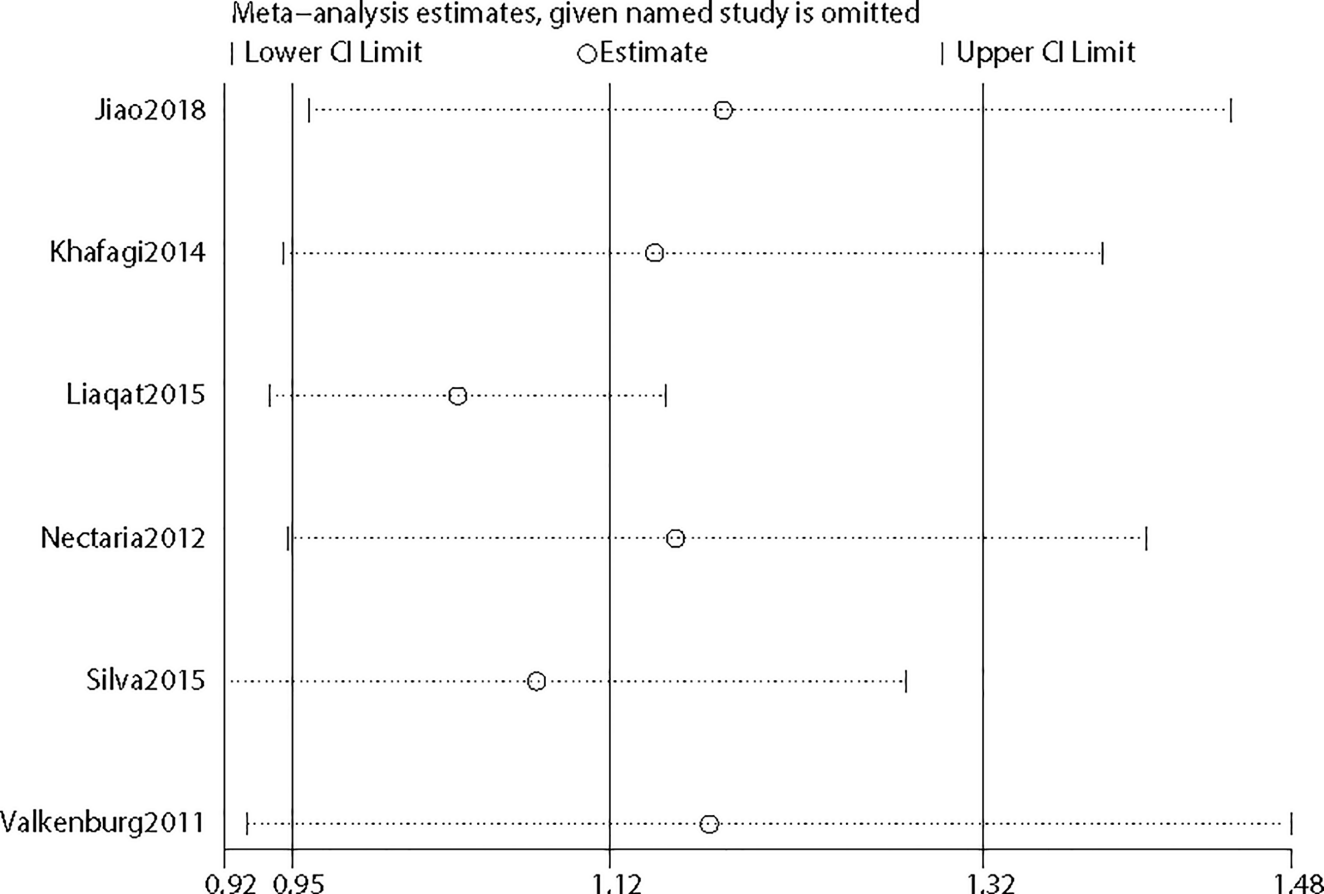
Sensitivity analysis of the included studies.

To further confirm the robustness of synthesized results, sensitivity analysis was conducted by removing each study once in five genetic models, respectively, and **Figure 5** depicts the pooled ORs and 95%CIs after deleting a single study in the allele model for rs2234693. For rs2234693, one study (16) in Asians contributed greatly to the high heterogeneity, as I^2^ decreased from 50% to 0% with a consistent result (OR=1.07 95%CI 0.98–1.18) after excluding this study. For rs9340799, the research of Jiao et al. (23) in Asians seemed to be the source of noticeable heterogeneity. With the removal of this study, the heterogeneity decreased from high (88%) to low (2%) in the Q test, and consistently, the result demonstrated no association between rs9340799 and PCOS susceptibility (OR=1.14 95%CI 0.97–1.34). By comprehensively scrutinizing these two pieces of research, ethnicity contributed remarkably to the inconsistency, and obviously, the results of subgroup analyses also supported that ethnicity led to the potential discrepancy.

### Publication Bias

**Figure 6:**
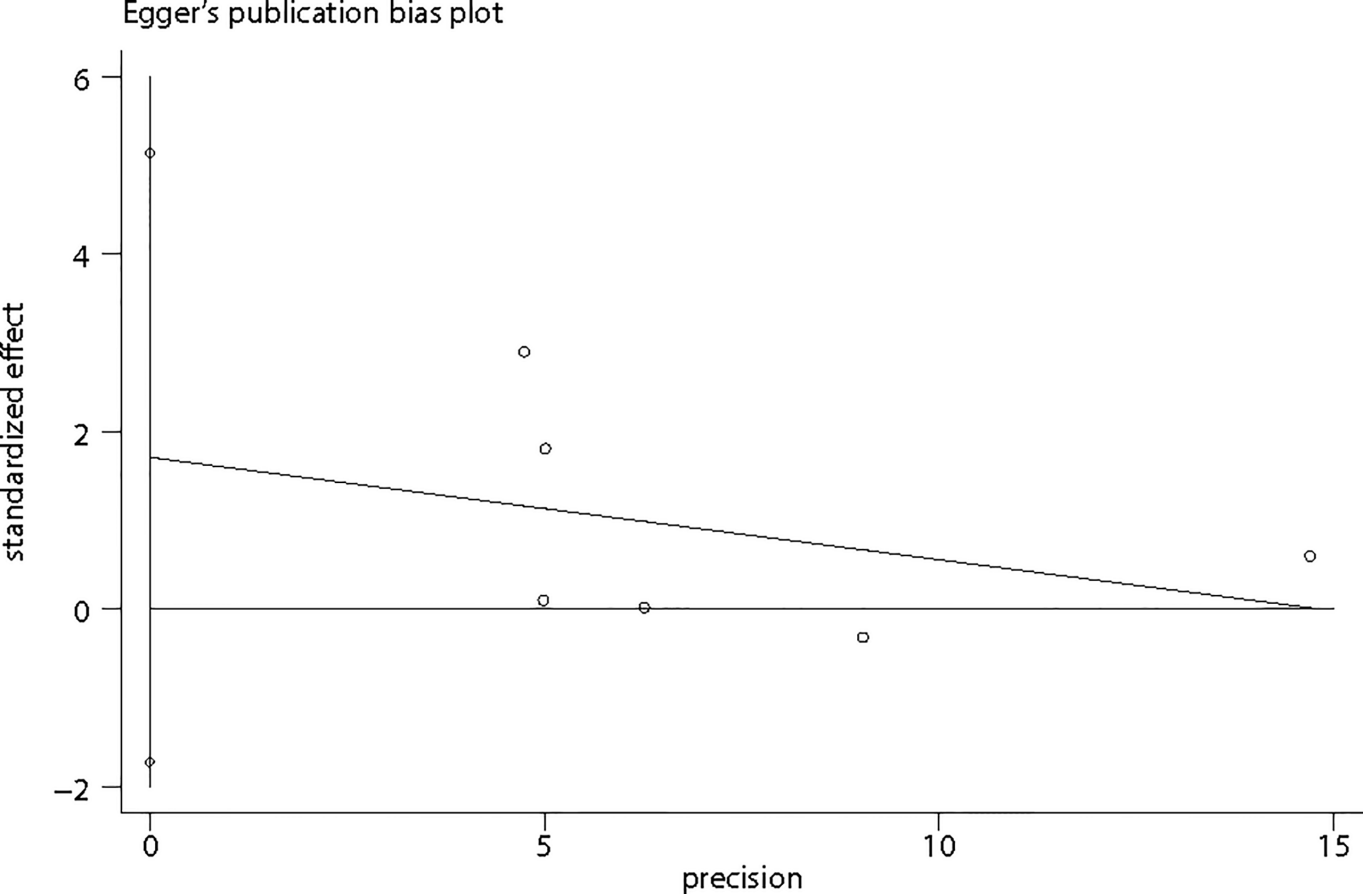
Egger’s publication bias plot.

Considering that the number of included studies was limited, it was inappropriate to apply Funnel plots to assess publication bias by visual inspection. Hence, Egger’s regression test was applied (**Figure 6**) to assess publication bias by formal statistic test. The P value for the Egger’s test was 0.238, indicating no potential publication bias among included studies. However, the results should be interpreted with caution due to the limited studies.

## Discussion

PCOS was generally considered implicated with genetic and hormonal factors. Accumulating shreds of evidence brought forward over 70 candidate genes associated with different genotypes of PCOS, especially in dysfunction of reproduction and endocrine metabolism (28). To our knowledge, this is the first and the most comprehensive meta-analysis concerning the correlation between ESR1/ESR2 polymorphisms and PCOS susceptibility. Our study found that rs2234693, rs9340799, and rs4986938 polymorphisms, the most extensively investigated in ESR1 and ESR2, might not play an essential role in PCOS susceptibility in the allele model, the dominant model, the recessive model, the heterozygote model, or the homozygote model from pooled analysis. Subgroup analyses according to the predefined factor of ethnicity indicated that these gene mutations were not associated with increased or decreased risk of PCOS in Asians and Caucasians separately. Furthermore, ethnicity might be the source of inconsistency due to low heterogeneity in subgroups. After sensitivity analysis by removing individual study once sequentially, the results remained consistent, which enhanced the robustness and liability of our conclusion.

Until now, at least 2,200 SNPs and 700 SNPs in ESR1 and ESR2 were identified (www.snpper.chip.org). Two best-characterized SNPs of rs2234693 and rs9340799 in the ESR1 are located in the first intron, which are 391 and 351 bp upstream of exon 2 and named as PvuII and XbaI, respectively (29). Also, the widely investigated rs4936938 polymorphism in the ESR2 is at position 1859 in the 3’-untranslated region of exon 8 and named as AluI (16). Although the transcriptional regulation attributed to these SNPs was not clarified adequately, feasible functional mechanisms were raised that they changed the expression of ESR1 and ESR2 by altering the binding of transcription factors (30). One previous human observational research manifested that there was markedly increased ESR1 expression in the endometrium tissue from PCOS women accompanied with a higher ESR1/ESR2 ratio than the comparable healthy group (31). Another study found similar results by isolating follicles and granulosa cells from PCOS women *versus* regular cycling women: the expression of ESR1 was higher in follicles, while the expression of ESR2 was lower in granulosa cells compared with the control group (32). Furthermore, these three SNPs were found to be associated with various estrogen-dependent diseases such as venous miscarriage, onset of menopause, and fracture risk after menopause (33–35).

Folliculogenesis and ovulation are complicated processes that involved a variety of hormone interactions. It is a typical feature of PCOS that dominant follicles fail to develop consistently, and previous research also observed that the concentration of estrogen was low in PCOS follicular fluid compared with that in dominant follicles (36). A biological function was clarified that estrogen can directly stimulate the proliferation of granulosa cells, attenuate apoptosis and follicular atresia, and increase the expression of hormone receptors (37, 38). Estrogen performed function of proliferation by binding to ERα expressed in the theca cells, while it also mediated the differentiation action and stimulated late follicle growth by attaching with ERβ localized in granulosa cells of developing follicles at all stages. Further studies also suggested that ERα and ERβ, which were both high-affinity ligand-dependent transcription factors (39, 40), were expressed not only in the ovaries but also in the hypothalamus. They played important roles in inducing gonadotropin-releasing hormone (GnRH) release as well as upregulating the expression of GnRH receptors in the anterior pituitary, thus promoting positive regulation of estrogen on the hypothalamus–pituitary axis and maintaining regular ovulation (41–43). This estrogen-ER complex recruited coregulatory proteins (coactivators or corepressors) to the promoter and regulated gene expression by binding to estrogen response element sequences in the promoter region of estrogen-responsive genes (44). Considering direct and indirect effects of estrogen on follicle development, maturation, and ovulation mediated by ERα and ERβ, polymorphisms of ESR1 and/or ESR2 would be expected to be associated with persistent anovulation in PCOS and an ocean of studies focused on genetic variations in ESR1 and ESR2 with pathological dysfunction of ovulation. However, there was a persistent inconsistency among studies concerning SNPs in the estrogen signaling pathway and susceptibility of PCOS. Our studies synthesized all of the current studies and solved the controversy by reporting no significant associations between the variants of ESR1 rs2234693, rs9340799, and ESR2 rs4936938 with PCOS.

One thing worth being called attention to is that in spite of the fact that no statistical significance was found, PCOS patients tended to have a higher ratio of rs2234693 polymorphism in ESR1 than control women, while rs9340799 and rs4986938 polymorphisms distributed equally in two groups according to our results. Furthermore, it was found in subgroup analysis that when the population was divided into different ethnicities, the range of 95%CI was wider than the ultimate meta-result of rs2234693. It showed that accumulating sample sizes led to a more obvious result tilting to the right, indicating that sample size might contribute greatly to the distribution of SNPs. Rs2234693 could still be a promising candidate SNP involved in the occurrence of PCOS, which needed more large sample-size studies to verify. Nevertheless, the conclusion we drew from this meta-analysis might be influenced by underpowered effect. It was well known that PCOS was a multifactorial syndrome, which meant single gene mutation might contribute little, while the interaction of multiple genes might play a decisive role in the susceptibility of the diseases. In the future, genetic association studies should focus on comprehensive genotyping on candidate gene loci, analyzing the majority of variations across the entire gene region by linkage disequilibrium. Meanwhile, the gene–gene and gene–environment interactions will also be the priorities in future studies on the etiology of PCOS.

Some superiorities need to be addressed in this meta-analysis. First, the included studies in pooled analysis were all of moderate or high quality according to the most widely accepted assessment tool for observational studies, the NOS system, which guaranteed the reliability of raw data. Second, although the number of studies was limited, Egger’s linear regression test suggested that no obvious publication bias was detected. Third, we searched all mainstream databases by comprehensive search strategies and references in eligible studies irrespective of language; thus, selection bias was controlled. Finally, the results of sensitivity confirmed the robustness of our conclusion after strict inclusion and exclusion criteria.

However, several limitations should be noticed when interpreting the results of our meta-analysis. First, the ESR1 and ESR2 genes were generally considered highly polymorphic, while only 20 SNPs were under detection for correlation with PCOS and only 3 widely investigated SNPs (rs2234693, rs9340799, and rs4936938) were detected in more than three studies. Due to the fact that numerous SNPs were reported merely once or twice, for which the results cannot be meta-analyzed, there might be other potential PCOS susceptibility loci. Furthermore, the pathogenesis of PCOS was complicated, which meant a single gene mutation was unlikely to remarkably influence the risk of the disease, while disequilibrium between SNPs in ESR genes might be a risk factor. Second, although eligible studies were conducted in different countries, raw data from Africans were unavailable. We tried to contact the corresponding author, but no response was received. As the distribution of SNPs in different ethnicities was conspicuously distinct, the results should be interpreted with caution. Third, PCOS was defined as a heterogeneous disease for its complicated phenotypes. Due to the absence of biochemical parameters of PCOS patients in eligible studies, subgroup stratification analyses based on phenotypes were unable to be performed. Hence, more well-designed studies with a large sample size and elaborate clinical data conducted in different ethnicities were strongly required.

## Conclusions

In conclusion, our systematic review and meta-analysis found that the rs2234693 and rs9340799 polymorphisms in the ESR1 gene and rs4936938 polymorphism in the ESR2 gene were not likely to be associated with individual PCOS susceptibility, even if ethnicity was taken into account. The conclusion we draw from the meta-analysis may guide and promote the study of pathogenesis and etiology of PCOS in gene–gene and gene–environment interactions.

## Data Availability Statement

The original contributions presented in the study are included in the article/supplementary material. Further inquiries can be directed to the corresponding author.

## Author Contributions

SZ contributed to the conception of the study. SZ and SW contributed to the selection of articles, evaluation of evidence quality, and extraction of data. SZ performed the data analyses and wrote the manuscript. SW, YS, MY, XS, YC, DK, and LX helped perform the analyses with constructive discussions. All authors contributed to the article and approved the submitted version.

## Funding

This project was supported by Scientific Research Projects of The National Natural Science Fund (81671421 and 81971354).

## Conflict of Interest

The authors declare that the research was conducted in the absence of any commercial or financial relationships that could be construed as a potential conflict of interest.

## Publisher’s Note

All claims expressed in this article are solely those of the authors and do not necessarily represent those of their affiliated organizations, or those of the publisher, the editors and the reviewers. Any product that may be evaluated in this article, or claim that may be made by its manufacturer, is not guaranteed or endorsed by the publisher.
